# Патогенетически обоснованное применение в реальной клинической практике вагинального эстриола 0,5 мг у женщин различных возрастов

**DOI:** 10.14341/probl13575

**Published:** 2025-05-20

**Authors:** Е. Н. Андреева, Е. В. Шереметьева, А. Ф. Веснина, Ж. А. Ужегова

**Affiliations:** Национальный исследовательский центр эндокринологии; Национальный исследовательский центр эндокринологии; Российский университет медицины; Национальный исследовательский центр эндокринологии; Национальный исследовательский центр эндокринологии

**Keywords:** эстриол, менопауза, атрофия влагалища, дефицит эстрогенов, аменорея, локальная терапия

## Abstract

сследования последних десятилетий показывают неуклонный рост средней продолжительности жизни человека, и женщин в частности. Любые эпителиальные ткани реагируют на изменение окружающей их гормональной среды сходным образом, но ни одна из них не может сравниться с эпителием свода влагалища и шейки матки по скорости и отчетливости реакции на гормоны, в первую очередь на половые стероиды. Урогенитальный тракт особенно чувствителен к снижению уровня эстрогенов, и около половины всех женщин, как в репродуктивном возрасте, так и в периоде гормональной перестройки, могут испытывать симптомы, связанные с вульвовагинальной атрофией, затрагивающие сексуальное здоровье и качество жизни. Эстриол — основной эстроген, который таргетно решает проблемы, обусловленные эстрогенной недостаточностью: диспареунии, сухости и зуда во влагалище и нижних отделах мочеполового тракта, нарушения мочевыведения, умеренного недержания мочи, а также рецидивирующего вульвовагинита и цистита. Согласно международным и российским клиническим рекомендациям патогенетически обоснованно назначение 0,5 мг с высоким уровнем убедительности и достоверности. Вульвовагинальная дистрофия у женщин различных возрастов — это мультидисциплинарная проблема на стыке гинекологии, урологии и дерматологии, которая может и должна быть решена для предотвращения более тяжелой гинекологической и урологической патологий.

## Abstract (en)

Research in recent decades has shown a steady increase in the average life expectancy of humans, and women in particular. Any epithelial tissue reacts to changes in the surrounding hormonal environment in a similar way, but none of them can compare with the epithelium of the vaginal vault and cervix in terms of the speed and clarity of reaction to hormones, primarily sex steroids. The urogenital tract is especially sensitive to a decrease in estrogen levels, and about half of all women, both in reproductive age and during hormonal changes, may experience symptoms associated with vulvovaginal atrophy, affecting sexual health and quality of life. Estriol is the main estrogen that specifically addresses problems caused by estrogen deficiency: dyspareunia, dryness and itching in the vagina and lower genitourinary tract, urinary disorders, moderate urinary incontinence, as well as recurrent vulvovaginitis and cystitis. According to international and Russian clinical guidelines, the prescription of 0.5 mg is pathogenetically justified with a high level of persuasiveness and reliability. Vulvovaginal dystrophy in women of different ages is a multidisciplinary problem at the intersection of gynecology, urology and dermatology, which can and should be solved to prevent more severe gynecological and urological pathologies.

Симптомы вульвовагинальной атрофии (ВВА), согласно мировой статистике, есть у 50% женщин в возрасте 55–80 лет, но только 11% из них будут говорить о ней как о жалобе и проблеме [1]. По данным по европейской популяции: один и более симптомов вульвовагальной атрофии имеют до 57,5% и не получают при этом никакого, в т.ч. патогенетического, лечения [2]. Интенсивность симптомов ВВА имеет прямую корреляцию со снижением качества жизни женщины в мено- и постменопаузе [3].

Не стоит забывать, что гипоэстрогенные состояния на уровне гениталий могут возникнуть у женщин любого возраста, в том числе связанные с гипоталамической аменореей или первичной недостаточностью яичников, и связаны с явлениями симптоматической ВВА. Факторами риска вульвовагинальной атрофии принято считать: менопаузу, двустороннюю овариэктомию, преждевременную недостаточность яичников, курение, злоупотребление алкоголем, половое воздержание и снижение половой активности, отсутствие родов через естественные родовые пути, другие причины низкого уровня эстрогена (например, послеродовой период, гипоталамическая аменорея), лечение онкологических заболеваний, включая лучевую терапию органов малого таза, химиотерапию и гормональную терапию [4].

Влагалище и шейка матки являются чувствительными индикаторами влияния эстрогенов, доступными для объективного осмотра. Чтобы понимать, что происходит с эпителием влагалища на фоне дефицита эстрадиола, необходимо знать структуру влагалища на морфологическом уровне. Стенка влагалища состоит из слизистой, мышечной и адвентициальной оболочек. Слизистая оболочка представлена неороговевающим многослойным плоским эпителием (МПЭ), его толщина достигает 25–40 рядов клеток в репродуктивном возрасте. В МПЭ выделяют 4 последовательных слоя клеток, начиная от основания: базальный, парабазальный, промежуточный и поверхностный. Базальный слой МПЭ расположен на базальной мембране и состоит из одного ряда маленьких округлых и низкоцилиндрических клеток с крупным овальным ядром, богатым хроматином. Эти клетки пополняют остальные слои МПЭ клеточными элементами и являются недифференцированными. Следующие 3 слоя клеток МПЭ различаются своей дифференцировкой. Парабазальный слой состоит из 2–3 рядов округлых или овальных клеток, которые крупнее базальных и содержат большое, занимающее почти половину клетки ядро, базофильную цитоплазму и немного гликогена. Клетки следующего, промежуточного и наиболее многорядного, слоя самые крупные, если сравнивать их с клетками других слоев, с небольшим ядром, но более выраженной светлой цитоплазмой с гликогеном. Они не способны к пролиферации и дифференцируются по мере их приближения к клеткам поверхностного слоя, при этом содержание гликогена в их цитоплазме возрастает, а сами клетки уплощаются. Отдельно необходимо остановиться на важности гликогена, синтезирование и накопление которого в большом количестве характерно именно для клеток МПЭ. Защитные функции регенерации и кератинизации соответствуют высокому уровню обмена в эпителии, а источником энергии для синтеза кератина является гликоген. Количество гликогена в МПЭ влагалища регулируется эстрадиолом. При его нормальном уровне происходит пролиферация МПЭ влагалища и повышение содержания гликогена в промежуточных клетках, с достижением максимума в фолликулярную фазу. Гликоген участвует в поддержании кислотности влагалища, т.к. имеется прямая связь между количеством гликогена и продукцией молочной кислоты. В результате десквамации и цитолиза поверхностных клеток МПЭ гликоген становится доступным для «питания» лактобактерий, которые, расщепляя его, выделяют молочную кислоту и спирты. Некоторые виды стрептококков, стафилококков, грамотрицательных бактерий и дрожжевых грибов, представляющих нормальную микрофлору здоровой женщины, тоже расщепляют гликоген, выделяя метаболиты, используемые лактобактериями для продукции кислоты. Концентрация молочной кислоты во влагалищном содержимом смещает рН среды в кислую сторону до 3,7–4,5. Такой уровень рН оптимален для жизнедеятельности нормальной микрофлоры влагалища, он тормозит рост и размножение микроорганизмов, чувствительных к кислой среде, проникающих из внешней среды или собственных условно-патогенных. На фоне гипоэстрогении клетки МПЭ уменьшаются в размере, и у промежуточного слоя снижается способность к синтезу гликогена. Дефицит гликогена приводит к анатомо-физиологической недостаточности МПЭ влагалища, сокращению количества лактобактерий и подъему уровня рН. Потеря этого защитного механизма делает эпителий влагалища уязвимым к инфекции и изъязвлениям, что, в свою очередь, снижает сексуальную уверенность женщины и вносит вклад в развитие диспареунии. И наконец, поверхностный (функциональный) слой МПЭ содержит крупные полигональные клетки с маленьким пикнотическим ядром и обильной эозинофильной цитоплазмой, где преобладают микрофиламенты кератина, гликогена же в этих клетках почти нет. Несмотря на наличие зерен кератогиалина в этих клетках МПЭ, полного их ороговения в норме не происходит [5]. Таким образом, МПЭ влагалища и влагалищной части шейки матки очень чувствителен к влиянию эстрогенов, под воздействием которых наблюдается:

1) усиление процессов пролиферации — в базальном и парабазальном слое МПЭ;

2) созревание клеток промежуточного слоя с накоплением в них гликогена;

3) созревание клеток поверхностного слоя с накоплением в них кератина [5][6].

Для фолликулярной фазы менструального цикла характерно выявление клеток поверхностного слоя, для лютеиновой — преимущественно промежуточных клеток, на фоне гипоэстрогении — базальных и парабазальных [4]. Из-за низкого уровня эстрадиола морфология МПЭ меняется и клетки больше не созревают, соответственно, могут отличаться высоким ядерно-цитоплазматическим соотношением, что может ошибочно интерпретироваться как цервикальная интраэпителиальная неоплазия [5][6].

На фоне дефицита эстрогенов происходят не только атрофические изменения слизистой влагалища, вульвы и уретры, но также меняется метаболизм и качество коллагена I и III типов, эластина, происходит их деструктуризация, вследствие чего влагалище утрачивает свою складчатость, уменьшается глубина и просвет вагинального канала, развивается опущение стенок влагалища. Уменьшение объема подслизистой сосудистой сети, ишемия стенки влагалища и, как следствие, уменьшение транссудации провоцируют жалобы на сухость и жжение во влагалище. Снижение пролиферативной активности базального и парабазального слоев МПЭ, истончение и малоэластичность атрофического эпителия делают его легкоранимым, вызывая жалобы на дискомфорт при коитусе и кровянистые выделения после, диспареунию, водянистые выделения из влагалища. Недостаточное созревание эпителия приводит к дефициту гликогена, снижению количества и элиминации лактобацил, смещению рН влагалища в щелочную сторону. Угнетение сопротивляемости тканей делает их уязвимыми для вторичной инфекции, что формирует почву для вторичной инфекции влагалища и мочевых путей. ВВА часто сопровождается урологическими симптомами, которые оказывают значительное влияние на качество жизни женщин. Они возникают из-за истончения слизистой уретры и пузыря, изменения качества коллагена и эластина, нарушения биоценоза влагалища. К сожалению, диагностика ВВА часто затруднена, т.к. женщины воспринимают все вышеперечисленные симптомы как естественные проявления старения и нечто неизбежное и не обращаются за помощью, перенося страдания молча — 77% женщин c симптомами ВВА считают неудобным обсуждать эти симптомы с врачом. Улучшение диагностики ВВА является первым шагом для своевременного подбора методов лечения ВВА с доказанной эффективностью и улучшения качества жизни женщины в постменопаузе [5][7].

## КЛИНИЧЕСКИЙ СЛУЧАЙ №1

Пациентка К., 31 год, направлена урологом к акушеру-гинекологу в связи с рецидивирующим циститом (обострения в течение последних 3 лет каждые 2–3 месяца), исключив бактериальный генез урологический патологии. Пациентка также предъявила жалобы на выраженную сухость при половой жизни, неэффективность лубрикантов на водной основе, жжение и неприятные ощущения во влагалище в течение нескольких дней после половой жизни. У пациентки К. в течение 4 лет наблюдается отсутствие менструации. Девушка работает тренером в спортзале, за последние годы ведет как групповые, так и индивидуальные занятия, ежедневно, без выходных. На фоне интенсивного графика работы отметила снижение веса за последние 3 года на 7 кг (ИМТ в настоящее время 17,4 кг/м²). Ограничивает себя в углеводсодержащей пище, разрешая себе «вкусные» продукты (хлеб, фрукты, твороженную запеканку и т.д.) только 1 раз в неделю. Не обследовалась у гинеколога в течение последних лет, отсутствие менструации в течение нескольких лет расценивала как благоприятные условия для работы. При обследовании женщины на фоне вторичной аменореи: по данным УЗИ органов малого таза, эндометрий — 3 мм, тонкий, однородный, строение яичников мультифолликулярное, объемы яичников — до 10 см³, гормональный анализ крови: ФСГ — 1,4 мМЕ/мл, ЛГ — 1,8 мМЕ/мл, эстрадиол — менее 50 пг/мл, пролактин и ТТГ — в рамках референсных значений, в биохимическом анализе крови обращает на себя внимание снижение общего белка, ферритин — 17 нг/мл, 25-ОН-D — 13 нг/мл, мазок на жидкостную онкоцитологию — атипических клеток не выявлено, атрофический тип мазка, по УЗИ молочных желез патологии не выявлено. При осмотре на гинекологическом кресле обращает на себя внимание: сухость, истончение кожи и слизистой вульвы и влагалища, при объективном обследовании методом кольпоскопии определяется истончение слизистой влагалища, кровоточивость, петехиальные кровоизлияния, многочисленные просвечивающиеся капилляры. Диагноз пациентки: «Дисфункция (гипогонадотропная) яичников репродуктивного периода (Е28.8) на фоне недостаточность массы тела» (ИМТ — 17,4 кг/м²). В рамках дообследования пациентке было рекомендовано проведение денситометрического исследования проксимального отдела бедренной кости и поясничного отдела позвоночника (согласно КР «Аменорея и олигоменорея» [8]). Помимо консультации диетолога, психоневролога (для исключения нарушения/расстройства пищевого поведения), ЗГТ в виде эстроген-гестагенного препарата, препарата колекальциферола и железа, пациентке было рекомендована локальная терапия эстриолом во влагалище в виде крема 0,5 мг, офф-лейбл1. В начале лечения локальный эстриол был назначен ежедневно в терапевтической дозе 0,5 мг в течение 2–4 недель (терапия насыщения), по мере улучшения — 2 раза в неделю длительно с последующей оценкой клинической картины симптоматически и повторной консультацией уролога. Через 1 месяц пациентка отметила практически полностью исчезновение дискомфорта, в т.ч. сухости, при половой жизни, отсутствие клинических симптомов обострения хронического цистита после половых контактов. В данном клиническом случает мы наблюдаем у пациентки с вторичным эстрогенодефицитом (функциональная гипоталомическая аменорея, дефицит массы тела) ВВА, которая сопровождается урологической патологией (рецидивирующей цистит неинфекционного генеза). Данная клиническая ситуация требует не только коррекции эстрогенодефицита системными комбинированными препаратами с половыми стероидами, но и таргетного применения эстриола.

## КЛИНИЧЕСКИЙ СЛУЧАЙ №2

Пациентка Н., 63 года, обратилась к гинекологу с жалобами на сухость во влагалище при половой жизни, жжение, зуд, частые позывы к мочеиспусканию после половой жизни в течение недели. Менопауза — с 51 года, выраженной вазомоторной симптоматики не отмечает. При общем осмотре: ИМТ — 30,2 кг/м², АД на обеих руках — 128–132/79–85 мм рт.ст., из хронических заболеваний женщины следует отметить: артериальную гипертензию 2 ст., 2 ст., сахарный диабет 2 типа (СД2), остеохондроз грудного и поясничного отделов позвоночника, постоянный прием лекарств: колекальциферол 2000 МЕ в день, метформин пролонгированного действия 1000 мг в день, дапаглифлозин 10 мг. При гинекологическом осмотре обращает на себя внимание сухость, истончение вплоть до атрофии кожи и слизистой вульвы и влагалища. При обследовании: маммография BIRADS 2, УЗИ органов малого таза — миома матки малого размера (1,2 см) с интерстициальным расположением узла, эндометрий 3 мм, однородный, яичники не определяются, по данным мазка на онкоцитологию — атрофического типа, атипические клетки не обнаружены, общий анализ мочи без патологических изменений. Диагноз пациентки Н., 63 года: «Генитоуринарный синдром. Постменопауза. Миома матки малого размера с интерстициальным расположением узла. ДДМЖ (BIRADS 2), СД2, ожирение I ст., артериальная гипертензия 2 ст., 2 ст.». Согласно КР «Менопауза и климактерическое состояние у женщин» [9], инструкции («заместительная гормональная терапия для лечения атрофии слизистой оболочки нижних отделов мочеполового тракта, связанной с эстрогеновой недостаточностью у женщин в постменопаузе») к препарату [10] — крем с эстриолом 0,5 мг, ежедневно в терапевтической дозе 0,5 мг в течение 2–4 недель (терапия насыщения), по мере улучшения — 2 раза в неделю длительно, основываясь на облегчении симптомов, до достижения поддерживающей дозы (т.е. 1 аппликация 2 раза в нед). Хотелось бы подчеркнуть, что согласно документу 2023 г. «Российские критерии приемлемости назначения менопаузальной гормональной терапии пациенткам с сердечно-сосудистыми и метаболическими заболеваниями. Согласительный документ РКО, РОАГ, РАЭ, ЕАТ, РАФ», назначение локального эстриола совместимо с сахароснижающими пероральными препаратами при наличии у пациентки СД2, также управляемая артериальная гипертензия не является противопоказаниям для локальной заместительной терапии [31]. Через 3 месяца на повторной консультации пациентка отметила выраженный положительный эффект: практически отсутствие сухости, жжения, зуда при половой жизни и после нее, снижение интенсивности позывов к мочеиспусканию. Пациентке было рекомендовано продолжить аппликации крема с эстриолом 0,5 мг 1 раз в неделю в течение 6 месяцев с последующей консультацией для оценки клинического эффекта и дальнейшей тактики ведения.

Идеальная профилактика ВВА у женщин старшей возрастной группы — это поддержание достаточного уровня эстрогенов в репродуктивном и пременопаузальном периодах, чего возможно достичь только в рамках персонифицированного консультирования [4]. В реальной клинической практике, к сожалению, мы это видим крайне редко, и речь идет уже необходимости не превентивной, а терапевтической медицины. Нужно отметить, что в настоящий момент терапия, как в рамках менопаузальной гормональной терапии (МГТ), так и местными эстрогенами, начинается крайне поздно (когда уже клиническая картина наиболее яркая) [4]. Генитоуринарный синдром в мено- и постменопаузе остается крайне недооцененной проблемой женщин старшей возрастной группы [11].

Согласно клиническим рекомендациям Международного общества по менопаузе (2020 г.), выбор терапии зависит от тяжести симптомов, эффективности и безопасности лечения для конкретного пациента и его потребностей. Негормональные методы лечения, доступные без рецепта, способствуют облегчению для большинства женщин при легком течении ВВА в менопаузе. Дозы вагинальных эстрогенов, вагинального ДГА, системная терапия эстрогенами и оспемифенов являются эффективными методами лечения умеренного и тяжелого течения ВВА в менопаузе. При введении вагинального эстрогена, ДГА или оспемифена прием гестагена не показан. В настоящее время недостаточно данных для подтверждения безопасности вагинального эстрогена, ДГА или оспемифена у женщин с ВВА в менопаузе и раком молочной железы (РМЖ) (необходима консультация онколога) [12][17]. Согласно европейского протоколу (2020 г.), золотым стандартом лечения ВВА в менопаузе является эстроген-содержащая терапия, использование которой рекомендуется при умеренных и тяжелых степенях ВВА в менопаузе, которые не купируются негормональными методами. Можно использовать в сочетании с негормональными методами лечения [11]. Согласно российскому протоколу (2020 г.), рекомендуемым методом лечения следует считать при отсутствии противопоказаний локальную гормональную терапию:

Протокол подчеркивает, что симптомы часто возвращаются после прекращения лечения [13].

Урологические и гинекологические рекомендации акцентируют внимание клинициста на дозировке эстриола 0,5 мг в разовой дозе (КР «Недержание мочи у женщин», 2020, КР, проект «Менопауза и климактерическое состояние у женщины», 2024 г.) [24][25], что доказано в ходе РКИ и систематических обзоров [26–30].

Большинство авторов и все клинические протоколы по ведению пациенток с ВВА в мено- и постменопаузе рекомендуют патогенетическое лечение — применение эстроген-содержащей терапии таргетно, т.е. интравагинально. Почему эстриол, а не другие эстрогены?

Еще с 90-х годов было показано, что эстриол, применяемый вагинально:

Одной из тенденций современной фармакологии является не столько получение новых биологических активных молекул или их модификаций, сколько создание новых фармакологических форм: микронизованных, нанонизованных, в виде МЧ, НЧ или «троянских» частиц, предназначенных для различного локального введения. По этим причинам микронизация труднорастворимых препаратов является наиболее подходящим решением. Микронизированные препараты могут входить в состав таблеток, спреев, мазей, кремов. Такие препараты используются для лечения широкого спектра заболеваний (кардиология, пульмонология, гинекология) [14–16]. Микронизация эстриола позволяет решать вопросы не только на уровне тканей вульвы и влагалища, но и оказывать протективный эффект на нижние отделы мочевой системы. Интравагинальное введение корректирует симптомы урогенитальной атрофии и симптомы недержания мочи, что отмечено при кольпоскопии и измерении уретрального давления [18]. Положительный эффект при урологической патологии отмечен в клинических рекомендациях 2024 г.: «Рекомендуется для уменьшения выраженности недержания мочи проводить пациенткам постменопаузального возраста с недержанием мочи вагинальную терапию эстриолом (в виде лекарственных форм для местного применения), при наличии симптомов вульвовагинальной атрофии» [19].

За счет каких же еще механизмов мы видим протективный таргентный эффект эстриола? Матричные металлопротеазы (ММТ 2 и 9) и катепсины ответственны за разрушение коллагена у женщин в постменопаузе. Увеличение дисфункции фибробластов — одна из важнейших причин влагалищной атрофии, и терапия местным эстрогеном направлена на восстановление выработки коллагена и подавления экспрессии MMT (к сожалению, не влияет при стрессовом недержании мочи). При сравнении различных схем лечения (ДГА, предшественники эстрогена, тестостерон, местный эстроген) было выявлено, что более высокая плотность коллагена наблюдалась именно на фоне терапии местными эстрогенами [20]. Следует помнить, что у женщин с выпадением влагалища происходит замена коллагена I типа на коллаген III типа, редифференцировка гладких мышц в миофибробласты и мышечные архитектурные изменения наблюдались в передней стенке влагалища. Потеря коллагена связана как с гипоэстрогенией, так и с генетическими факторами [21–22].

Согласно размещенному 02.08.2024 г. на сайте Государственного реестра лекарственных средств обновленному (дополненному) перечню взаимозаменяемых лекарственных препаратов, опираясь на государственный документ (Взаимозаменяемые лекарственные препараты (п. 4 ч. 4 ст. 3 Федерального закона от 27.12.2019 № 475-ФЗ, п. 8 постановления правительства от 05.09.2020 № 1360)), не все доступные в Российской Федерации лекарственные препараты, суппозитории вагинальные, в которых 1 суппозиторий содержит эстриол 0,5 мг, являются взаимозаменяемыми референтному лекарственному препарату Овестин, суппозитории вагинальные, в котором 1 суппозиторий содержит эстриол микронизированный 0,5 мг [23]. Эти важные аспекты важно и нужно учитывать лечащему врачу для достижения максимальной эффективности в лечении и, конечно же, безопасности проводимой терапии [4].

Таким образом, механизм положительного влияния эстриола при интравагинальном использовании заключается в следующих процессах:

## ЗАКЛЮЧЕНИЕ

Вульвовагинальная атрофия — одно из наиболее драматичных и нежелательных проявлений дефицита эстрогенов. 77% женщин считают неудобным обсуждать ее симптомы с врачом. Повышение качества диагностики этого состояния является первым шагом для своевременного назначения лечения с доказанной эффективностью и улучшения качества жизни женщины на фоне гипоэстрогении, в т.ч. в постменопаузе. Хотя официальная распространенность атрофии влагалища меняется в зависимости от численности и индивидуальных особенностей изучаемого населения, все большее число женщин страдают от этого состояния по мере старения населения. ВАА является хроническим и прогрессирующим состоянием. Своевременное информирование пациентов о причинах возникновения вышеуказанных симптомов и возможностях их устранения может позволить в короткие сроки улучшить состояние женщин, вернуть им интерес к жизни и повысить ее качество.

## ДОПОЛНИТЕЛЬНАЯ ИНФОРМАЦИЯ

Источники финансирования. Работа выполнена в рамках государственного задания №123021300169-4 «Эпигенетические предикторы и метаболомная составляющая аменореи различного генеза у женщин репродуктивного возраста», 2023–2025 гг.

Конфликт интересов. Авторы декларируют отсутствие явных и потенциальных конфликтов интересов, связанных с содержанием настоящей статьи.

Участие авторов. Все авторы одобрили финальную версию статьи перед публикацией, выразили согласие нести ответственность за все аспекты работы, подразумевающую надлежащее изучение и решение вопросов, связанных с точностью или добросовестностью любой части работы.

1. Офф-лейбл (англ. off-label, от off — за пределами, label — этикетка, инструкция) — использование лекарственных средств по показаниям, не утвержденным государственными регулирующими органами, не упомянутым в инструкции по применению, т.е. вне клинического протокола.
